# The effects of probiotic and synbiotic supplementation on metabolic syndrome indices in adults at risk of type 2 diabetes: study protocol for a randomized controlled trial

**DOI:** 10.1186/s13063-017-1885-8

**Published:** 2017-03-29

**Authors:** Nazila Kassaian, Ashraf Aminorroaya, Awat Feizi, Parvaneh Jafari, Masoud Amini

**Affiliations:** 1 Isfahan Endocrine and Metabolism Research Center, Isfahan University of Medical Sciences, Isfahan, Iran; 2 Biostatistics and Epidemiology Department, School of Health, Cardiac Rehabilitation Research Center, Isfahan Cardiovascular Research Institute, Isfahan University of Medical Sciences, Isfahan, Iran; 3 Microbiology Departments, Science Faculty, Islamic Azad University (IAU), Arak Branch, Arak, Iran

**Keywords:** Probiotic, Synbiotic, Impaired glucose tolerance, Metabolic syndrome, Microbiome

## Abstract

**Background:**

The incidence of type 2 diabetes, cardiovascular diseases, and obesity has been rising dramatically; however, their pathogenesis is particularly intriguing. Recently, dysbiosis of the intestinal microbiota has emerged as a new candidate that may be linked to metabolic diseases. We hypothesize that selective modulation of the intestinal microbiota by probiotic or synbiotic supplementation may improve metabolic dysfunction and prevent diabetes in prediabetics. In this study, a synthesis and study of synbiotics will be carried out for the first time in Iran.

**Methods/Design:**

In a randomized triple-blind controlled clinical trial, 120 adults with impaired glucose tolerance based on the inclusion criteria will be selected by a simple random sampling method and will be randomly allocated to 6 months of 6 g/d probiotic, synbiotic or placebo. The fecal abundance of bacteria, blood pressure, height, weight, and waist and hip circumferences will be measured at baseline and following treatment. Also, plasma lipid profiles, HbA1C, fasting plasma glucose, and insulin levels, will be measured and insulin resistance (HOMA-IR) and beta-cell function (HOMA-B) will be calculated at baseline and will be repeated at months 3, 6, 12, and 18. The data will be compared within and between groups using statistical methods.

**Discussion:**

The results of this trial could contribute to the evidence-based clinical guidelines that address gut microbiota manipulation to maximize health benefits in prevention and management of metabolic syndrome in prediabetes.

**Trial registration:**

Iranian Registry of Clinical Trials: IRCT201511032321N2. Registered on 27 February 2016.

**Electronic supplementary material:**

The online version of this article (doi:10.1186/s13063-017-1885-8) contains supplementary material, which is available to authorized users.

## Background

Diabetes mellitus (DM), prediabetes, obesity, and cardiovascular disorders (CVD) are important public health problems in that their prevalence and incidence have been rising dramatically during the last decades [1]. The prevalence of prediabetes in the world exceeds 25% who are at higher risk of developing type 2 diabetes (T2DM), compared with individuals with normal glucose tolerance [2].

In addition to well-established risk factors for T2DM and CVD, including genetic predisposition and unhealthy lifestyle, an altered configuration of the microbial community in the gut has emerged as a new candidate that may be linked to these metabolic disorders [2, 3]. Studies suggest that aspects of the modern Western lifestyle, including high-fat diets and antibiotics, can alter commensal microbial communities. However, a full list of factors which are capable of altering gut microbiota remains incomplete [4]. Studies have shown that early intervention in patients with impaired glucose tolerance can significantly prevent type 2 diabetes [3]. Recent researches suggest that intestinal microbiota (i.e., Bacteria, Archaea and Eukarya residing in the gastrointestinal tract) are associated with low-grade chronic inflammation and may play a role in the pathogenesis of obesity- and cardiovascular disease -related events [5, 6]. The role of gut microbiota in metabolic disorders may be through influence on host nutrition and energy, intestinal epithelial homeostasis, and host immune system [7]. It is shown that endotoxin concentrations are higher in adults at risk of T2DM compared with normoglycemic individuals and are associated with an increased risk of insulin resistance, CVD, and incidence of type 2 diabetes [8, 9].

The distal gut microbiota is composed of billions of bacteria, of which Bacteroidetes and Firmicutes are the two dominant phylogenetic types [10]. Firmicutes are the largest phylum of bacteria, comprising more than 200 genera, and the Bacteroidetes include over 20 bacterial genera [11]. Most of the studies in humans and in mice have reported that obesity and impaired glucose metabolism are associated with an altered ratio of Firmicutes and Bacteroidetes [12, 13].

It is documented that the ratio of Bacteroidetes to Firmicutes is positively and significantly correlated with plasma glucose concentrations [8, 14]. In a study in mice, obesity has been associated with a 50% reduction in Bacteroidetes and increase in Firmicutes [15].

An approach for modulation in the gut microbiota is the use of oral viable strains of bacteria (probiotics), indigestible carbohydrates (prebiotics), or synergistic combinations of a probiotic and prebiotic (synbiotic) [16, 17].

Based on previous studies, prebiotics result in specific changes in the activity and/or composition of the gut microbiota mostly in rodent models, which can be beneficial for local and systemic host health. Most health aspects of prebiotics have been directed toward obesity and inflammation [18]. Furthermore, probiotic supplementation has been shown to reduce metabolic endotoxemia and improve glycemic control in rodents [19]. Therefore, it is hypothesized that prebiotic and/or probiotic supplementation may be a useful strategy to improve metabolic health, insulin resistance, and prevent type 2 diabetes, which could be interesting in the management of metabolic disorders in prediabetics. To establish the causality, well-designed and well-powered prospective studies in which an altered microbiome is documented during supplementation with probiotic, prebiotic or synbiotic are needed [20]. In this field, the evidence is limited and the results are inconsistent. The discrepancies between studies can be explained by ethnic or dietary differences. Also, knowledge on the long-term efficacy of these treatments and comparison of their effects in a randomized clinical trial is still lacking, especially in the human. The intervention period of most of the previous trials has been too short (less than 3 months) to observe the effects of these supplements. In addition, there are limited studies which explored the effects of probiotic or synbiotic administration in prediabetic individuals. Up to now, there has been no clinical trial to assess the effects of these supplements on insulin resistance and risk of diabetes development in patients with prediabetes.

Hence, this study has been designed to investigate the efficacy of probiotic and synbiotic administration in the development of type 2 diabetes and metabolic variables in prediabetics via a parallel-group randomized triple-blind placebo-controlled clinical trial.

## The aims and hypotheses

### General aim

The general aim of the present investigation is to determine the effects of 6 months ingestion of probiotic or synbiotic on metabolic syndrome indices and gut microbiota composition through a triple-blind, randomized, placebo-controlled clinical trial in adults at risk of type 2 diabetes (prediabetes).

### The specific aims and hypotheses

#### Aim 1

To determine whether probiotic or synbiotic supplementation improves fasting plasma glucose, insulin resistance (primary outcome), and beta-cell function in individuals at elevated risk of developing T2DM. We hypothesize that probiotic and synbiotic supplementation will improve fasting plasma glucose, insulin sensitivity, and beta-cell function in these individuals compared with placebo.

#### Aim 2

To determine whether probiotic or synbiotic supplementation will improve lipid profiles in individuals at elevated risk of developing T2DM. We hypothesize that probiotic and synbiotic supplementation will reduce serum triglyceride and non-high-density lipoprotein (HDL) cholesterol, elevate HDL cholesterol, and improve low-density lipoprotein (LDL)/HDL, total cholesterol (TC)/HDL and triglyceride (TG)/HDL in these individuals.

#### Aim 3

To determine whether probiotic or synbiotic supplementation will improve anthropometric indicators in individuals at elevated risk of developing T2DM. We hypothesize that probiotic and synbiotic supplementation will improve body mass index (BMI), waist circumference and waist-to-hip ratio (WHR) in these individuals.

#### Aim 4

To determine the changes in abundance and ratio of two dominant groups of gut microbiota including Firmicutes and Bacteroidetes and their relation with metabolic syndrome indices during supplementation. We hypothesize that the change in Gram-positive gut microbiota (Firmicutes) and Firmicutes-to-Bacteroidetes ratio with probiotic and synbiotic supplementation will be correlated with the change observed in the metabolic variables.

#### Aim 5

To compare the effects of probiotic and synbiotic supplements on gut microbiota, anthropometric, glycemic, and lipid profiles. We hypothesize that synbiotic supplements will have more efficacy to improve these indicators than probiotic.

## Methods/Design

### Overview

A parallel-group randomized triple-blind placebo-controlled clinical trial, designed according to the CONSORT SPIRIT 2013 guidelines (Additional file 1), will be conducted over a period of 1.5 years from June 2016, in Isfahan, Iran. We will include 120 prediabetic adults [individuals with impaired glucose tolerance (IGT) or impaired fasting glucose (IFG)], as determined by the American Diabetes Association (ADA) criteria [21]. The participants will be selected from first-degree family of type 2 diabetic patients. They will be recruited from the outpatient clinic of Isfahan Endocrine and Metabolism Research Center.

Eligibility criteria for the participants are presented in Table 1. A nutritionist will perform the intervention. The participants will be randomized into three equal groups using a blocking stratified sampling method by age and sex to ensure equal numbers within each age and sex strata using a computerized table of random digits. The groups are probiotic, synbiotic (6 grams inulin + probiotic) or placebo (maltodextrin) consumers for 6 months. The appearance and packaging of the three products are identical. Details about the content and preparation process of pruductes will be presented in section 3.2. The inulin dose of 6 g/d as the prebiotic in the synbiotic type was selected based on previous studies [22]. This dose was suggested and tested to be well-tolerated. Participants, laboratory staff, and investigators will be blinded to the allocation of probiotic, synbiotic or placebo, which will be conducted by the study pharmacist (who will then dispense blinded supplements or placebo to the investigators). The subjects will be instructed to dilute the powder in water and to drink it after their main meal to minimize the killing of the probiotic by gastric acid. Due to the dietary-controlled nature of the study, water was selected as the beverage for the delivery of supplements. Individuals will be appropriately blinded in performing outcome measurements and type of supplement. The supplements will be assigned letter A, B or C and are otherwise identical. All of the participants have been counseled to follow an energy-balanced diet and physical activity recommendations 6 months previous to start of the study. They will be advised not to modify their daily dietary and physical activity habits. To ensure that these habits have not been modified during the experiment, the participants will be instructed to record daily food and physical activity diaries, which will be checked monthly during face-to-face visits by a dietitian. Also, the participants will be advised not to modify their medication during the experiment. During these visits, the researchers will check the compliance with the study supplementation and the development of any adverse events. Compliance will be assessed based on returned tablet counts. The project coordinator will assess and manage solicited and spontaneously reported adverse events of trial interventions.

**Table 1:** Eligibility criteria for the effects of probiotic or synbiotic supplementation on metabolic syndrome indices

Inclusion criteria:	
Population: men and women 35–75 years old	
Prediabetic [FBG = 100–125 OR 2hBS = 140–199 (via OGTT)]	
Informed consent	
Exclusion criteria:	
Current smokers, suspected or definite history of alcohol or drug abuse history	
Antibiotic use in the past 3 months or during the treatment period	
Using probiotic, prebiotic or symbiotic during the past 3 months	
Noncompliance in consumption of the supplements	
Being pregnant	
Having gastrointestinal diseases, i.e., food allergies, celiac, irritable bowel disease,	
severe liver, kidney, heart, nervous system diseases	
Allergy to studied agents	
Currently taking prescribed nonsteroidal anti-inflammatory drugs, antipsychotics, nicotinic acid	

*FBG* fasting blood glucose, *2hBS* 2-hour blood sugar, *OGTT* oral glucose tolerance test

**Fig. 1:**
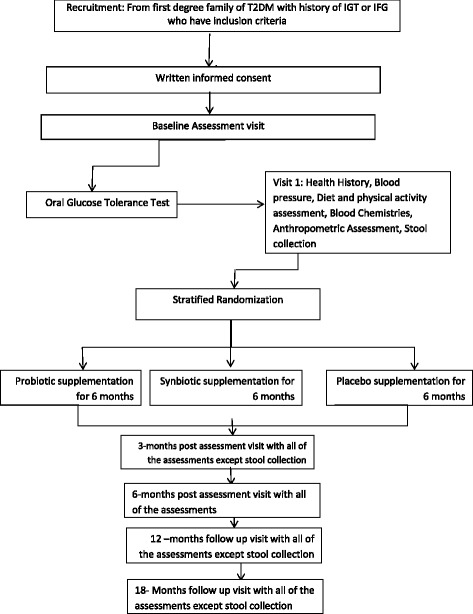
Study design schematic

**Fig. 2:**
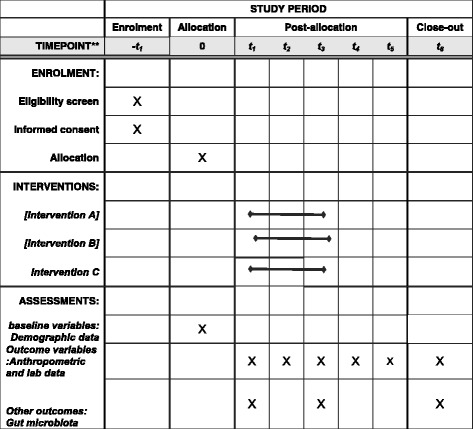
Template of content for the schedule of enrollment, interventions, and assessments

If a participant will be found to have missed >10% of supplement dose at follow-up, he or she will be excluded from the study. Also, the participants will be excluded from the study if there is any change in medication, they start antibiotics, or modify their lifestyle. The framework of the study has been provided in Fig. 1. Study visits will be scheduled for weeks 1, 2, 3, 5, 7, 9, 12, 14, 16, 18, 20, 24, 48, and 72. A table of the time schedule of enrollment, interventions, assessments, and visits for participants is provided in Table 2, and an overview of the timeline of the study interventions and assessments is provided in Fig. 2.

**Table 2:** Time schedule of enrollment, interventions, assessments, and visits for participants

Week	Visits and activities
Prior to starting the study	Patients who meet eligibility criteria will be asked to read and sign the consent form
Week 1	Patients will be instructed on writing the food and physical activity records and procedures for collection of stool samples. They will be asked not to take any other dietary supplementation or probiotics and not to change their medication and/or lifestyle while in the study and to inform the researchers if they are prescribed oral antibiotics at any time during the study
Week 2	Stool samples, food record and physical activity record will be collected. Weight, height, waist and hip circumferences, and blood pressure will be measured. Biochemical tests will be performed. The supplement will be delivered
Week 3, 5, 7, 9	Contact with the participants and effectiveness or adverse event will be recorded.
Week 12	Food record and physical activity record will be collected. Weight, height, waist and hip circumferences, and blood pressure will be measured. Biochemical tests will be performed. The supplement will be delivered
Week 14, 16, 18, 20	Contact with the participants and effectiveness/adverse event will be recorded
Week 24	Stool samples, food record and physical activity record will be collected. Weight, height, waist and hip circumferences, and blood pressure will be measured. Biochemical tests will be performed
Week 48, 72	Food record and physical activity record will be collected. Weight, height, waist and hip circumferences, and blood pressure will be measured. Biochemical tests will be performed

### Product standardization and palatability testing

Participants will be supplemented with 6 g/d of either synbiotics comprising a probiotic with an inulin-based prebiotic, probiotics containing the freeze-dried *Lactobacillus acidophilus*, *Bifidobacter bifidum*, *Bifidobacter lactis*, and *Bifidobacter longum* (10^9^ for each) with maltodextrin as filler, or placebo including maltodextrin for 6 months.

The supplements were prepared and packaged in Tak Gen Zist Pharmaceutical Company, Tehran, Iran in sachet form.

All of microbial and purity tests were checked by two independent microbiologists.

Prior to the onset of the trial, the solubility and palatability of supplements (6 g dose) in a cup of water were assessed to determine the optimal well-tolerated mode of delivery of the supplement prescription.

The researchers will be in weekly contact with participants and any concerns or side effects during the intervention period will be addressed. The participants will be excluded from the study if there is any side effect or problem.

### Biochemical assays

Participants who have met the inclusion criteria (Table 1) will be instructed to arrive at Isfahan Endocrine and Metabolism Research Center for laboratory testing between 7:00 and 9:00 am after a 12-hour overnight fast and not to do vigorous physical activity for the previous 48 hours. Each participant will be tested for the biochemical parameters at baseline and during each follow-up visit (3, 6, 12, and 18 months).

An oral glucose tolerance test (OGTT) will be performed to clarify glycemic status. Plasma lipid and lipoprotein concentrations (i.e., total cholesterol, high- and low-density lipoprotein cholesterol, and triglycerides) will be measured using a photometric assay kit (Pars Azmoon Co., Tehran, Iran). Fasting glucose levels will be measured using the glucose oxidase (GOD) method. Fasting insulin levels will be measured using chemiluminescence (Siemens, Munich, Germany). Ion exchange chromatography will be used to test glycosylated hemoglobin (HbA1c).

The homeostatic model assessment of insulin resistance (HOMA-IR) and homeostatic model assessment of beta-cell function (HOMA-B) index will be used to determine insulin resistance (primary outcome) and beta-cell function respectively using the following formula:

$$ \mathrm{HOMA}\hbox{-} \mathrm{I}\mathrm{R}=\mathrm{fasting}\;\mathrm{blood}\;\mathrm{glucose}\;\left(\mathrm{mmol}/\mathrm{ml}\right)\times \mathrm{insulin}\;\mathrm{assay}\;\left(\mathrm{microunit}/\mathrm{ml}\right)/22.5 $$

$$ \upbeta \kern0.5em \mathrm{cell}\kern0.5em \mathrm{function}\kern0.5em \left(\mathrm{HOMA}\hbox{-} \mathrm{B}\right)=\left[\mathrm{fasting}\ \mathrm{insulin} \times 20\right]/\left[\mathrm{fasting}\ \mathrm{glucose}\hbox{-} 3.5\right] $$

### Anthropometric parameters and blood pressure measurement

Height, weight, waist and hip circumferences will be measured at baseline and will be repeated at months 3, 6, 12, and 18.

Height will be determined using a scale-mounted stadiometer to the nearest 0.5 cm and weight will be measured with light clothing to the nearest 0.1 kg. BMI will be calculated by dividing each participant’s weight in kilograms by height in meters squared. Waist circumference will be measured at the midpoint between the lowest rib and the iliac crest to the nearest 0.1 cm using a flexible tape. Hip circumference will be measured around the widest portion of the buttocks by the tape. The waist-to-hip ratio (WHR) will be calculated according to World Health Organization (WHO) recommendations [23]. All the measurements will be taken by one person to decrease the error rate.

Blood pressure for each participant will be measured by a trained nurse on two occasions using a mercury sphygmomanometer in both of the hands and the mean blood pressure readings will be used for the analysis according to American Heart Association guidelines [24]. Participants will be instructed to rest for 10 minutes prior to the first blood pressure measurement with a minimum of 15 minutes between the two occasions.

### Assessment of physical activity and dietary intake

Food and physical activity records will be assessed at five time points during the study (at months 0, 3, 6, 12, and 18). At each time point, participants will be instructed to record their daily food and beverage intake for 3 days, including a weekend day. To enhance portion size accuracy, food scales and models will be also used. The portion sizes will be converted to grams, and every food and beverage item will be subsequently coded and analyzed for energy content, macro and micro nutrients using Nutritionist 4 [25].

Physical activity will be assessed using the metabolic equivalent of task (MET) questionnaire [26].

### Assessment of gut microbiota

Fecal samples will be obtained daily for 3 days at baseline and at the final 3 days of the intervention (after 6 months) in sterile plastic containers and delivered to the Infectious Diseases and Tropical Medicine Research Center Laboratory within 4 hours of collection. After being given a code, samples will be immediately frozen at −80 °C until final processing. DNA will be extracted from fecal samples (from the 3 composite days mixed equally) using DNA Extraction Stool Mini Kit (Qiagen, Hilden, Germany) according to the manufacturer’s instructions. Numbers of phylum Firmicutes and Bacteroidets will be determined by quantitative real-time polymerase chain reactions (qPCR) methodology using specific designed primers and Taqman probes.

The composition and ratio of the two fecal bacteria before and after supplementation with probiotics, synbiotics, and placebo will be compared.

## Data analysis

### Sample size

Calculation of sample size was based on a parallel three-group randomized clinical trial with repeated measurements of main outcomes at three time points. Considering a type one error rate of $\upalpha =0.05$, and statistical power of 1 − β = 0.80 (^*Z*^_1 − *β*_ = 0.84) for detecting a standardized effect size of at least Δ = 0.60 in order to have the largest number of participants in a repeated measures design structure based on the following formula, 29 subjects were determined. Compensating for possible attrition, 30% additional samples will be recruited, in which a final 40 subjects in each study group will participate.

$$ R=\left[\frac{1+\left( w-1\right)\;\rho}{w}-\frac{v{\rho}^2}{1+\left( v-1\right)\rho}\right];{n_{1\;}}_{repeated}= R\left[\frac{2{\left({z}_{1-\alpha /2}+{z}_{1-\beta}\right)}^2}{\varDelta^2}+\frac{z_{{}_{1-\alpha /2}}^2}{4}\right] $$

In this formula, Δ is the standardized effect size, R will be determined based on number of observations before intervention, v (in our study v = 1), number of observation after intervention, w (in the current study w = 2) and intracluster correlation coefficient ρ, that was considered as ρ = 0.7 [27].

The participant’s adhesion and retention will be managed by the project coordinator who will conduct weekly contact. We anticipate that it will take 12 months to recruit this sample size. All of the outcome data will be collected even for participants who discontinue or deviate from intervention protocols.

### Randomization

A computer-generated list of random numbers is used to create a series of sequentially numbered envelopes containing equal assignments to either placebo, probiotic or symbiotic. The study epidemiologist is responsible for the randomization and the study pharmacist for delivery of the blinded supplements. The supplements, which will be assigned letter A, B or C, are otherwise identical and the participants, investigators, outcome assessors, and data analysts will be blinded after assignment to interventions.

### Statistical analysis

Record data including outcome data and adverse events will be double-entered on SPSS software (Version [15], SPSS Inc., Chicago, IL, USA) to secure its validity. Descriptive univariate analyses on all study variables through presenting them as mean ± standard deviation (SD) (for normally distributed continuous) or median [interquartile range (IQR)] for nonnormal quantitative data and frequency (percentage) for categorical data will be conducted. Data will be evaluated for the presence of outliers, violations of normality, and missing data. Major violations of normality will be corrected with an appropriate transformation procedure, i.e., logarithmic transformation for positively skewed and exponential function for negatively skewed data. In the case of an outlier, rather than transform the data, the outlier will be “Winsorized,” that is, replaced by the most extreme value in the tail of the distribution as well as a sensitivity analysis being performed when outliers are in or out of analyses. To test our hypothesis that probiotic and synbiotic supplementations will improve main outcomes in prediabetic participants, a repeated measures analysis of variance (ANOVA) with a between-subject factors approach will be used. The multiple observations will be considered as nested data within individuals. This will allow us to make inference about time effects (i.e., comparison between the time points while accounting for the correlation in the data) in each studied treatment group and to make inference about differences between competing treatment groups. This approach enables us to make an inference regarding the group by time interaction as a primary important interested component. As a perquisite, the assumption of sphericity will be evaluated using a Muchly test, in the case of violation, multivariate or Huynh-Feldt approaches will be employed. A compound symmetry error structure will be chosen for this model when the assumption is present. We will use repeated measures ANOVA as the main approach for testing all our hypothesis regarding the main outcomes; simultaneously, independent *t* test along with Bonferroni adjustment will be conducted for comparing outcomes in each follow-up time point between study groups. Some prespecified subgroup analysis will be done based on some confounding variables such as age, gender, etc., for detecting significant differences in intervention efficacy across the strata of mentioned variables. However, these subsamples will be compared to the main dependent variables at baseline. If the groups are found to be different from one another then they will be entered into the model as a covariate. On the other hand, the baseline values of the main dependent variables and other potential confounding variables will be considered as covariates and they will be adjusted in all fitted models. The study analyst will have access to the results and final trial dataset and make the final decision on interim analyses and terminate the trial if there is any harm for the participants.

### Treatment of missing data

Missing data are common in longitudinal studies. Based on patterns and the mechanism of missing data, an appropriate multiple imputation approach will be followed for completing missing data. We will use an intention-to-treat analysis as our primary analytic approach. As an alternative statistical analysis method using linear mixed model ANOVAs or generalized estimating equation (GEE) for longitudinal analysis, we will compare main study outcomes across the treatment groups. Through employing these methods, the possibility for using all available data to construct weighted averages across the different patterns of missing data to provide valid point estimates, and confidence intervals for population parameters will be provided [28]. As a secondary analysis, we will conduct a per protocol analysis and restrict the analyses to only those individuals who complete the intervention; if the results differ we will interpret the findings based on the intention-to-treat analysis.

## Discussion

Up to now, the therapeutic effects of gut microbiota manipulation, through the administration of probiotics/prebiotics/synbiotics, have been discussed, but there is little information regarding if they improve metabolic indicators in humans. In addition, the potential benefits of these biopharmaceuticals in prediabetic individuals who are at high risk for developing type 2 diabetes and experiencing cardiovascular events has received little attention. This trial will address this important research gap, by exploring and comparing the role of probiotics and synbiotics in modifying anthropometric, glycemic, and lipid profiles among prediabetic individuals.

The innovative aspects of this clinical trial include: (1) testing hypotheses involving a novel concept for which there are little strong data in humans; (2) focusing on individuals at high risk for T2DM, including those with prediabetes; (3) carrying out a synthesis and study of synbiotics for the first time in Iran; (4) following up the participants for 1 year after the intervention phase; and (5) comparing the effects of probiotics and synbiotics.

Importantly, our findings could have a significant impact on clinical practice and public health as a simple and efficacious adjunctive approach for prevention or management of T2DM if our hypotheses are supported. The primary challenge of our study will be participant adhesion and retention. This challenge will be managed by the project coordinator who will have weekly contact with participants in which any concerns or issues will be immediately addressed.

If these supplements are shown to be effective in management of prediabetes and preventing diabetes, they would be a very attractive alternative or adjunctive for current medications.

### Trial status

Estimated enrollment is 120 participants.

The study start date was June 2016, and estimated study completion date is June 2018.

## Additional file

Additional file 1: SPIRIT 2013 Checklist: recommended items to address in a clinical trial protocol and related documents. (DOCX 76 kb)

## Abbreviations

ANOVA: Analysis of variance
BMI: Body mass index
CVD: Cardiovascular disease
HbA1c: Glycosylated hemoglobin
HDL: High-density lipoprotein
HOMA-B: Homeostatic model assessment of beta-cell function
HOMA-IR: Homeostatic model assessment of insulin resistance
LDL: Low-density lipoprotein
qPCR: Quantitative real-time polymerase chain reaction
T2DM: Type 2 diabetes mellitus
WHR: Waist-to-hip ratio
